# Evaluation of Altmetric Analysis Scores of the Top 100 Articles on Polycystic Ovary Syndrome Published in the Last 10 Years

**DOI:** 10.7759/cureus.32903

**Published:** 2022-12-24

**Authors:** Mehmet Mete Kırlangıç, Erdem Sahin, Mefkure Eraslan Sahin, Serhan Kütük, Belfin Nur Arıcı Halıcı, Mehmet Ak

**Affiliations:** 1 Obstetrics and Gynaecology, Kartal Dr Lutfi Kirdar City Hospital, Istanbul, TUR; 2 Obstetrics and Gynaecology, Kayseri City Hospital, Kayseri, TUR; 3 Obstetrics and Gynaecology, Develi State Hospital, Kayseri, TUR; 4 Obstetrics and Gynaecology, Tuzla State Hospital, Istanbul, TUR

**Keywords:** social media, trend topics, traditional metrics, altmetric score, pcos

## Abstract

Objective: In the current study, we analyzed the 100 most cited articles with the topic, title, and keywords of polycystic ovary syndrome (PCOS) published in all journals in terms of traditional metrics and the altmetric score (AS).

Methods: The term “polycystic ovary syndrome (PCOS)” was searched in the Web of Science (WoS) database and filtered for articles published in all journals. Bibliographic data and AS were obtained for 100 highly cited papers from January 2012 to July 2022. Descriptive statistics were reported and correlation analysis between traditional bibliographies and the AS was performed.

Results: The Journal of Clinical Endocrinology & Metabolism, with 14 articles, had the most publications on the Top 100 list. When the studies were classified according to subtypes, 56 papers were original scientific papers with mean AS of 32.5 (15.3-52.7), whereas 44 papers were reviews and meta-analyses with AS of 16.0 (8.6-43.2). The AS was positively correlated with H-index, total WoS citation number of article and Q category. There were no correlations with impact factor (IF), five-year IF, journal impact factor (JIF) percentile and journal citation indicator (JCI) value.

Conclusion: Our results suggest that the AS is related with article total WoS citation number, journal Q category, and journal H-index. Effective communication on social media can promote scientific productivity and have a positive impact on society.

## Introduction

Polycystic ovary syndrome (PCOS) is the most common endocrine disorder in reproductive age women and patients are adversely affected due to metabolic, reproductive, and psychological impacts [1]. Obesity, metabolic syndrome, type 2 diabetes, and an increased risk of cardiovascular disease are well-known important complications of PCOS and remain the most common cause of anovulatory infertility today [2-4]. The diagnosis of PCOS is obtained after excluding other causes and by detecting two of the existing Rotterdam criteria (biochemical/clinical hyperandrogenism, oligo-amenorrhea, and polycystic ovaries on ultrasound) [5,6].

In the scientific world, the quality of research is determined by the number of citations and the impact factor of the journal. Today, the use of social media is growing rapidly, and this situation is becoming increasingly important for academic literature and scientific journals. Altmetrics is an alternative assessment of research driven by social media that includes the number of views, downloads, and reads of an article on social media and mainstream media and inclusion in public policy documents, in addition to the citation number, and measures the impact of the article online. Altmetrics was designed to monitor and quantify the impact of research based on social media mentions and collects dissemination data from social media channels such as Facebook, Twitter, Google+, etc. [7,8]. It is clear that the use of social media will increase in the future, and one-third of people in developed countries use social media today [9]. Thus, it is a natural progression of this widespread use of social media to include dissemination via social media and non-traditional sources in new citation analyses for reputable journals. Altmetric.com is one of the most popular websites that calculates altmetrics and offers them to researchers. All authors, including us, wonder how much impact our publications will have on social platforms. The altmetric system easily and practically provides authors with this information. As altmetrics increase in popularity, journals are beginning to provide altmetric scores in addition to citation scores for published research.

In this study, we used traditional metrics and altmetrics to analyze the 100 most cited articles about PCOS using the topic, title, and keywords published in all journals.

## Materials and methods

In this descriptive study, we selected the Web of Science (WoS) core collection database and searched using the topic, title, and keywords for “polycystic ovary syndrome and PCOS” in the basic search section. Journals in the “all medicine” category published from 01.01.2012 to 01.07.2022 were included. The resulting articles were sorted from highest to lowest citation number. The top 100 highly-cited articles on PCOS were included in the study (access date: 01.07.2022). Inclusion criteria for the articles included: imaging methods, diagnosis and treatment methods, etiology, epidemiology, pathophysiology, follow-up, and prognosis of PCOS. Topics, titles, and keywords unrelated to PCOS were not included. Title, first author, year of publication, total WoS citation number of article, journal H-index, journal impact factor (IF), journal 5-year impact factor (5-year IF), journal impact factor (JIF) percentile, journal Q category and journal citation indicator (JCI) value were evaluated and recorded. The "Altmetric it!" bookmarklet was used to obtain the altmetric score (AS) of the articles. This site provides the article's AS and a colored donut. Each color of the donut represents a different source that disseminated the article.

The Shapiro-Wilk test of normality was used to evaluate numerical data. The data was not normally distributed, so the median and 25%-75% interquartile range were used. Correlations between AS and other parameters were assessed using Spearman’s rank correlation. Percentages or numbers were used to present categorical data. SPSS software version 22 (IBM Corp., Armonk, NY, USA) was used for statistical analysis. A value of p<0.05 was accepted as significant.

## Results

According to the WoS search, 14,421 articles related to the topic, title, and keywords of PCOS were published between January 2012 and July 2022. The number of WoS citations of the papers listed in the Top 100 (T100) list ranged from 882 to 100 (Table 1).

**Table 1:** Top 100 article list. Note: WoS: Web of science, AS: Altmetrics score

No	Title		Journal	First author	Year	WoS database total cited	Times Cited in All Databases	AS
1	Diagnosis and treatment of polycystic ovary syndrome: an Endocrine Society clinical practice guideline	The Journal of Clinical Endocrinology & Metabolism		Legro, Richard S	2013	882	973	121
2	Insulin resistance and the polycystic ovary syndrome revisited: an update on mechanisms and implications	Endocrine Reviews		Diamanti-Kandarakis, Evanthia	2012	862	920	46
3	Consensus on women’s health aspects of polycystic ovary syndrome (PCOS): the Amsterdam ESHRE/ASRM-Sponsored 3rd PCOS Consensus Workshop Group.	Fertility and Sterility		Fauser, Bart CJM	2012	854	926	58
4	The prevalence and phenotypic features of polycystic ovary syndrome: a systematic review and meta-analysis	Human Reproduction		Bozdag, Gurkan	2016	492	527	25
5	Epidemiology, diagnosis, and management of polycystic ovary syndrome	Clinical Epidemiology		Sirmans, Susan M.	2014	478	506	157
6	Polycystic ovary syndrome	Nature Reviews Disease primers		Azziz, Ricardo	2016	464	478	72
7	The pathogenesis of polycystic ovary syndrome (PCOS): the hypothesis of PCOS as functional ovarian hyperandrogenism revisited.	Endocrine Reviews		Rosenfield, Robert L.	2016	440	470	43
8	Polycystic ovary syndrome: definition, aetiology, diagnosis and treatment.	Nature Reviews Endocrinology		Escobar-Morreale, Héctor F	2018	436	476	44
9	Overweight, obesity and central obesity in women with polycystic ovary syndrome: a systematic review and meta-analysis	Human Reproduction		Lim, Siew S.	2012	382	414	20
10	Scientific statement on the diagnostic criteria, epidemiology, pathophysiology, and molecular genetics of polycystic ovary syndrome	Endocrine Reviews		Dumesic, Daniel A	2015	378	407	98
11	Criteria, prevalence, and phenotypes of polycystic ovary syndrome	Fertility and Sterility		Lizneva, Daria	2016	362	400	65
12	Women with polycystic ovary syndrome have intrinsic insulin resistance on euglycaemic–hyperinsulaemic clamp	Human Reproduction		Stepto, Nigel K.	2013	362	388	25
13	Genome-wide association study identifies eight new risk loci for polycystic ovary syndrome	Nature Genetics		Shi, Yongyong	2012	359	383	5
14	Fresh versus frozen embryos for infertility in the polycystic ovary syndrome	New England Journal of Medicine		Chen, Zi-Jiang	2016	351	395	281
15	Prevalence, phenotype and cardiometabolic risk of polycystic ovary syndrome under different diagnostic criteria	Human Reproduction		Yildiz, Bulent Okan	2012	346	368	5
16	Recommendations from the international evidence-based guideline for the assessment and management of polycystic ovary syndrome	Human Reproduction		Teede, Helena J.	2018	334	359	60
17	The polycystic ovary syndrome: a position statement from the European Society of Endocrinology	European Journal of Endocrinology		Conway, Gerard	2014	332	377	9
18	Letrozole versus clomiphene for infertility in the polycystic ovary syndrome	New England Journal of Medicine		Legro, Richard S	2014	290	319	283
19	Circulating markers of oxidative stress and polycystic ovary syndrome (PCOS): a systematic review and meta-analysis	Human Reproduction Update		Murri, Mora	2013	268	281	5
20	The effect of obesity on polycystic ovary syndrome: a systematic review and meta‐analysis	Obesity Reviews		Lim, S. S.	2013	265	287	28
21	Definition and significance of polycystic ovarian morphology: a task force report from the Androgen Excess and Polycystic Ovary Syndrome Society	Human Reproduction Update		Dewailly, Didier	2014	243	266	6
22	Epidemiology, diagnosis and management of hirsutism: a consensus statement by the Androgen Excess and Polycystic Ovary Syndrome Society.	Human Reproduction Update		Escobar-Morreale, H. F.	2012	241	260	9
23	Risk of endometrial, ovarian and breast cancer in women with polycystic ovary syndrome: a systematic review and meta-analysis	Human Reproduction Update		Barry, John A	2014	234	248	43
24	Insulin‐sensitising drugs (metformin, rosiglitazone, pioglitazone, D‐chiro‐inositol) for women with polycystic ovary syndrome, oligo amenorrhoea and subfertility.	Cochrane Database of Systematic Reviews		Tang, Thomas	2012	232	236	0
25	Pregnancy complications in women with polycystic ovary syndrome	Human Reproduction Update		Palomba, Stefano	2015	225	241	12
26	The management of anovulatory infertility in women with polycystic ovary syndrome: an analysis of the evidence to support the development of global WHO guidance.	Human Reproduction Update		Balen, Adam H.	2016	223	246	38
27	Inflammation in polycystic ovary syndrome: underpinning of insulin resistance and ovarian dysfunction	Steroids		González, Frank	2012	263	262	17
28	Prevalence of polycystic ovary syndrome in women in China: a large community-based study	Human Reproduction		Li, Rong	2013	214	304	0
29	American Association of Clinical Endocrinologists, American College of Endocrinology, and androgen excess and PCOS society disease state clinical review: guide to the best practices in the evaluation and treatment of polycystic ovary syndrome-part 1.	Endocrine Practice		Goodman, Neil F.,	2015	212	258	33
30	American Association of Clinical Endocrinologists, American College of Endocrinology, and Androgen Excess and PCOS Society disease state clinical review: guide to the best practices in the evaluation and treatment of polycystic ovary syndrome-part 2.	Endocrine Practice		Goodman, Neil F.,	2015	204	230	6
31	Identification of microRNAs in human follicular fluid: characterization of microRNAs that govern steroidogenesis in vitro and are associated with polycystic ovary syndrome in vivo	The Journal of Clinical Endocrinology & Metabolism		Sang, Qing	2013	203	226	7
32	Genome-wide association of polycystic ovary syndrome implicates alterations in gonadotropin secretion in European ancestry populations	Nature communications		Hayes, M. Geoffrey	2015	202	210	70
33	Rodent models for human polycystic ovary syndrome	Biology of reproduction		Walters, Kirsty A	2012	201	211	2
34	High prevalence of moderate and severe depressive and anxiety symptoms in polycystic ovary syndrome: a systematic review and meta-analysis	Human Reproduction		Cooney, Laura G.	2017	196	200	10
35	Interactions between androgens, FSH, anti-Müllerian hormone and estradiol during folliculogenesis in the human normal and polycystic ovary.	Human Reproduction Update		Dewailly, Didier	2016	194	207	1
36	The potential implications of a PCOS diagnosis on a woman’s long-term health using data linkage	The Journal of Clinical Endocrinology & Metabolism		Roger Hart	2015	189	203	67
37	Polycystic ovary syndrome	New England Journal of Medicine		McCartney, Christopher R.	2016	188	205	157
38	Cardiometabolic aspects of the polycystic ovary syndrome.	Endocrine Reviews		Randeva, Harpal S	2012	178	184	3
39	Causal mechanisms and balancing selection inferred from genetic associations with polycystic ovary syndrome	Nature communications		Day, Felix R	2015	172	177	136
40	Hyperandrogenemia predicts metabolic phenotype in polycystic ovary syndrome: the utility of serum androstenedione	The Journal of Clinical Endocrinology & Metabolism		O'Reilly, Michael W.	2014	172	185	15
41	Can anti-Müllerian hormone predict the diagnosis of polycystic ovary syndrome? A systematic review and meta-analysis of extracted data	The Journal of Clinical Endocrinology & Metabolism		Iliodromiti, Stamatina	2013	172	185	3
42	Longitudinal weight gain in women identified with polycystic ovary syndrome: results of an observational study in young women	Obesity		Teede, Helena J.	2013	170	183	35
43	Metformin and lifestyle modification in polycystic ovary syndrome: systematic review and meta-analysis	Human Reproduction Update		Naderpoor, Negar	2015	169	180	34
44	Elevated prenatal anti-Müllerian hormone reprograms the fetus and induces polycystic ovary syndrome in adulthood	Nature medicine		Tata, Brooke	2018	168	177	415
45	miRNA-93 inhibits GLUT4 and is overexpressed in adipose tissue of polycystic ovary syndrome patients and women with insulin resistance	Diabetes		Chen, Yen-Hao	2013	164	181	29
46	Obstetric complications in women with polycystic ovary syndrome: a systematic review and meta-analysis	Reproductive Biology and Endocrinology		Qin, Jun Z.	2013	164	176	11
47	Characterization of reproductive, metabolic, and endocrine features of polycystic ovary syndrome in female hyperandrogenic mouse models	Endocrinology		Caldwell, A. S. L	2014	160	172	0
48	Is PCOS an inflammatory process?	Fertility and Sterility		Duleba, Antoni J.	2012	159	165	16
49	Adipose tissue dysfunction, adipokines, and low-grade chronic inflammation in polycystic ovary syndrome	Reproduction		Spritzer, Poli Mara	2015	158	174	2
50	Vitamin D in the aetiology and management of polycystic ovary syndrome.	Clinical endocrinology		Thomson, Rebecca L.	2012	156	169	8
51	The prevalence of polycystic ovary syndrome in a normal population according to the Rotterdam criteria versus revised criteria including anti-Müllerian hormone.	Human Reproduction		Lauritsen, M. P.	2014	152	167	15
52	Genetic, hormonal and metabolic aspects of PCOS: an update	Reproductive Biology and Endocrinology		De Leo, V.	2016	151	165	10
53	Gut microbiota–bile acid–interleukin-22 axis orchestrates polycystic ovary syndrome.	Nature medicine		Qi, Xinyu	2019	149	166	136
54	Sheep models of polycystic ovary syndrome phenotype	Molecular and cellular endocrinology		Padmanabhan, Vasantha	2013	146	149	3
55	Large-scale genome-wide meta-analysis of polycystic ovary syndrome suggests shared genetic architecture for different diagnosis criteria	PLoS genetics		Day, Felix	2018	145	148	218
56	Polycystic ovary syndrome with hyperandrogenism is characterized by an increased risk of hepatic steatosis compared to nonhyperandrogenic PCOS phenotypes and healthy controls, independent of obesity and insulin resistance	The Journal of Clinical Endocrinology & Metabolism		Jones, Helen	2012	141	147	8
57	Polycystic ovary syndrome is a risk factor for type 2 diabetes: results from a long-term prospective study	Diabetes		Gambineri, Alessandra	2012	141	146	62
58	Insulin resistance in polycystic ovary syndrome: a systematic review and meta-analysis of euglycaemic–hyperinsulinaemic clamp studies	Human Reproduction		Cassar, Samantha	2016	139	149	2
59	The diagnosis of polycystic ovary syndrome during adolescence	Hormone research in paediatrics		Witchel, Selma F.	2015	136	141	10
60	An international consortium update: pathophysiology, diagnosis, and treatment of polycystic ovarian syndrome in adolescence	Hormone research in paediatrics		Ibáñez, Lourdes	2017	135	141	21
61	Divergences in insulin resistance between the different phenotypes of the polycystic ovary syndrome	The Journal of Clinical Endocrinology & Metabolism		Moghetti, Paolo	2013	135	138	2
62	Delayed diagnosis and a lack of information associated with dissatisfaction in women with polycystic ovary syndrome	The Journal of Clinical Endocrinology & Metabolism		Gibson-Helm, Melanie	2017	129	133	322
63	Replication of association of DENND1A and THADA variants with polycystic ovary syndrome in European cohorts	Journal of medical genetics		Goodarzi, Mark O	2012	127	135	4
64	Dysbiosis of gut microbiota associated with clinical parameters in polycystic ovary syndrome	Frontiers in Microbiology		Liu, Rui	2017	126	144	17
65	Alterations in gut microbiome composition and barrier function are associated with reproductive and metabolic defects in women with polycystic ovary syndrome (PCOS): a pilot study.	PloS one		Lindheim, Lisa	2017	126	153	48
66	11-Oxygenated C19 steroids are the predominant androgens in polycystic ovary syndrome	The Journal of Clinical Endocrinology & Metabolism		O’Reilly, Michael W.	2017	125	126	25
67	Metabolic risk in PCOS: phenotype and adiposity impact.	Trends in Endocrinology & Metabolism		Moran, Lisa J	2015	124	133	3
68	Metformin improves pregnancy and live-birth rates in women with polycystic ovary syndrome (PCOS): a multicenter, double-blind, placebo-controlled randomized trial	The Journal of Clinical Endocrinology & Metabolism		Morin-Papunen, Laure	2012	123	133	9
69	Polycystic Ovary Syndrome	Obstetrics & Gynecology		Azziz, Ricardo MD, MPH	2018	120	134	8
70	The prevalence of polycystic ovary syndrome in reproductive-aged women of different ethnicity: a systematic review and meta-analysis	Oncotarget		Ding, Tao	2017	120	134	24
71	Overexpression of a DENND1A isoform produces a polycystic ovary syndrome theca phenotype	Proceedings of the National Academy of Sciences of the United States of America		McAllister, Jan M.	2014	120	150	28
72	Cardiovascular and metabolic profiles amongst different polycystic ovary syndrome phenotypes: who is really at risk?	Fertility and Sterility		Daan, Nadine MP	2014	120	131	10
73	Evaluating the association between endometrial cancer and polycystic ovary syndrome	Human Reproduction		Haoula, Zeina	2012	119	133	7
74	Polycystic ovary syndrome (PCOS), an inflammatory, systemic, lifestyle endocrinopathy	Journal of steroid biochemistry and molecular biology		Patel, S	2018	118	126	5
75	Insulin‐sensitising drugs (metformin, rosiglitazone, pioglitazone, D‐chiro‐inositol) for women with polycystic ovary syndrome, oligo amenorrhoea and subfertility	Cochrane Database of Systematic Reviews		Morley, Lara C.	2017	118	124	0
76	Placental steroidogenesis in pregnant women with polycystic ovary syndrome	European Journal of Obstetrics & Gynecology and Reproductive Biology		Maliqueo, Manuel	2013	117	120	0
77	Altered microRNA and gene expression in the follicular fluid of women with polycystic ovary syndrome	Journal of assisted reproduction and genetics		Roth, Lauren W	2014	115	127	6
78	Metabolic profiles characterizing different phenotypes of polycystic ovary syndrome: plasma metabolomics analysis	BMC medicine		Zhao, Yue	2012	115	127	11
79	Roles of oxidative stress in polycystic ovary syndrome and cancers	Oxidative medicine and cellular longevity		Zuo, Tao	2016	114	123	1
80	Lifestyle modification programs in polycystic ovary syndrome: systematic review and meta-analysis	The Journal of Clinical Endocrinology & Metabolism		Domecq, Juan Pablo	2013	113	118	3
81	Cancer risk and PCOS	Steroids		Dumesic, Daniel A.	2013	113	124	33
82	Dietary composition in the treatment of polycystic ovary syndrome: a systematic review to inform evidence-based guidelines	Journal of the Academy of Nutrition and Dietetics		Moran, Lisa J.	2013	113	116	106
83	Emotional distress is a common risk in women with polycystic ovary syndrome: a systematic review and meta-analysis of 28 studies	Human Reproduction Update		Veltman-Verhulst, Susanne M.	2012	112	113	9
84	Effects of polycystic ovary syndrome (PCOS), sex hormones, and obesity on circulating miRNA-21, miRNA-27b, miRNA-103, and miRNA-155 expression	The Journal of Clinical Endocrinology & Metabolism		Murri, Mora	2013	111	118	3
85	Effects of myo-inositol in women with PCOS: a systematic review of randomized controlled trials."	Gynecological Endocrinology		Unfer, Vittorio	2012	111	120	8
86	Ethnicity, obesity and the prevalence of impaired glucose tolerance and type 2 diabetes in PCOS: a systematic review and meta-regression	Human Reproduction Update		Kakoly, N. S.	2018	109	121	0
87	Gut Microbial Diversity in Women With Polycystic Ovary Syndrome Correlates With Hyperandrogenism	The Journal of Clinical Endocrinology & Metabolism		Torres, Pedro J.	2018	109	124	153
88	Variants in DENND1A are associated with polycystic ovary syndrome in women of European ancestry	The Journal of Clinical Endocrinology & Metabolism		Welt, Corrine K	2012	108	115	1
89	Geographical Prevalence of Polycystic Ovary Syndrome as Determined by Region and Race/Ethnicity	International journal of environmental research and public health		Wolf, Wendy M.	2018	107	108	118
90	Enhancement of a robust arcuate GABAergic input to gonadotropin-releasing hormone neurons in a model of polycystic ovarian syndrome	Proceedings of the National Academy of Sciences of the United States of America		Moore, Aleisha M.	2015	107	110	4
91	Reproductive and Metabolic Phenotype of a Mouse Model of PCOS	Endocrinology		Leonie, E.	2012	107	107	3
92	Carotid artery intima-media thickness in polycystic ovary syndrome: a systematic review and meta-analysis	Human Reproduction Update		Meyer, Michelle L.	2012	106	109	8
93	Serum vitamin D levels and polycystic ovary syndrome: a systematic review and meta-analysis	Nutrients		He, Chunla	2015	104	113	17
94	Increased prevalence of anxiety symptoms in women with polycystic ovary syndrome: systematic review and meta-analysis	Fertility and Sterility		Dokras, Anuja	2012	104	117	42
95	Association between polycystic ovary syndrome and the risk of pregnancy complications	Medicine		Yu, Hai-Feng	2016	103	107	4
96	Dysbiosis of Gut Microbiota (DOGMA) – A novel theory for the development of Polycystic Ovarian Syndrome	Medical hypotheses		Tremellen, Kelton	2012	103	127	30
97	In vitro maturation as an alternative to standard in vitro fertilization for patients diagnosed with polycystic ovaries: a comparative analysis of fresh, frozen and cumulative cycle outcomes	Human Reproduction		Walls, M. L.	2015	102	105	25
98	In vitro maturation or in vitro fertilization for women with polycystic ovaries? A case–control study of 194 treatment cycles.	Fertility and Sterility		Gremeau, Anne-Sophie	2012	102	104	56
99	Laparoscopic drilling by diathermy or laser for ovulation induction in anovulatory polycystic ovary syndrome	Cochrane Database of Systematic Reviews		Farquhar, Cindy	2012	102	108	0
100	Poly Cystic Ovarian Syndrome: An Updated Overview	Frontiers in physiology		El Hayek, Samer	2016	100	110	15

The most cited article was "Diagnosis and treatment of polycystic ovary syndrome: an Endocrine Society clinical practice guideline" written by Richard S Legro, which was published in the Journal of Clinical Endocrinology & Metabolism in 2013 [6]. The highest AS was 415 for the article titled "Elevated prenatal Anti-Müllerian hormone reprograms the fetus and induces polycystic ovary syndrome in adulthood" written by Brooke Tata published in Nature Medicine in 2018 [10]. With 14 articles, the Journal of Clinical Endocrinology & Metabolism had the most publications on the Top 100 list. The New England Journal of Medicine journal had the highest IF and H-index (Table 2).

**Table 2:** Journal list of the Top 100 articles. IF: Impact Factor, JCI: Journal Citation Indicator, JIF: Journal Impact Factor, AS: Altmetrics Score, Q category: Quartile category

Journal Name	Number of Articles	2021-IF	JCI		5 Year IF	H index	JIF Percentile	Q category		Citation Number of articles	AS of Articles
The Journal of Clinical Endocrinology & Metabolism	14	5,958	1,32		6,793	363	78,42	1		193,71	52,78
Human Reproduction Update	11	17,179	3,2		19,417	190	99,41	1		193,09	15
Human Reproduction	11	6,353	2,22		7,736	236	90	1		258	17,63
Fertility and Sterility	6	7,49	2,18		8,109	217	93,53	1		283,5	41,16
Endocrine Reviews	4	25,261	2,96		27,899	283	97,6	1		464,5	47,5
New England Journal of Medicine	3	176,079	22,26		125,115	1079	99,13	1		276,33	240,33
Cochrane Database of Systematic Reviews	3	12,008	1,34		11,956	219	89,24	1		150,66	0
Biology of Reproduction	1	4,161	1,19		4,522	188	69,35	2		201	2
BMC Medicine	1	11,15		2,09	11,129	155	88,08	1	115		11
Clinical Endocrinology	1	3,523		0,7	3,822	153	36,64	3	156		8
Clinical Epidemiology	1	5,814		1,23	5,221	63	78,33	1	478		157
Diabetes	2	9,337		1,79	10,509	345	92,12	1	152,5		45,5
Endocrine Practice	2	3,701		0,77	4,072	91	43,49	3	208		19,5
Endocrinology	2	5,051		0,99	5,074	267	68,15	2	133,5		1,5
European Journal of Endocrinology	1	6,558		1,32	6,805	155	81,82	1	332		9
European Journal of Obstetrics & Gynecology and Reproductive Biology	1	2,831		0,68	2,778	104	46,47	3	117		0
Frontiers in Microbiology	1	6,064		98	6,843	166	75,37	1	126		17
Frontiers in Physiology	1	4,755		0,98	5,316	122	75,98	1	100		15
Gynecological Endocrinology	1	2,277		0,6	2,237	65	12,67	4	111		8
Hormone Research in Paediatrics	2	4,275		0,83	3,914	93	54,45	2	135,5		15,5
International journal of environmental research and public health	1	4,614		0,93	4,798	138	64,34	2	107		118
Journal of Assisted Reproduction and Genetics	1	3,357		0,92	3,763	81	47,71	3	115		6
Journal of Medical Genetics	1	5,954		1,41	6,455	177	84,29	1	127		4
Journal of Steroid Biochemistry and Molecular Biology	1	5,011		0,9	4,649	133	63,01	2	118		5
Journal of the Academy of Nutrition and Dietetics	1	5,234		1	6,524	181	71,67	1	113		106
Medical hypotheses	1	4,411		0,77	3,143	94	53,6	2	103		30
Medicine	1	1,817		39	2,227	90	29,36	3	103		4
Molecular and Cellular Endocrinology	1	4,369		0,74	4,467	152	44,59	3	146		3
Nature Communications	2	17,694		3,13	17,763	410	92,47	1	187		103
Nature Genetics	1	41,307		8,81	39,321	597	99,14	1	359		5
Nature Medicine	2	87,241		12,6	68,31	576	99,83	1	158,5		275,5
Nature Reviews Disease primers	1	65,038		13,47	83,065	128	97,38	1	464		72
Nature Reviews Endocrinology	1	47,564		5,35	47,776	165	99,17	1	436		44
Nutrients	1	6,706		1,09	7,185	143	83,89	1	104		17
Obesity	1	9,298		1,62	6,918	209	90,75	1	170		35
Obesity Reviews	1	10,867		1,61	12,19	172	94,18	1	265		28
Obstetrics & Gynecology	1	7,623		1,92	7,767	231	94,71	1	120		8
Oncotarget	1	NA		NA	NA	NA	NA	NA	120		24
Oxidative Medicine and Cellular Longevity	1	7,31		0,73	8,427	114	71,91	2	114		1
PLoS Genetics	1	6,02		1,34	6,514	244	84,86	1	145		218
PloSOne	1	3,752		0,88	4,069	367	60,96	2	126		48
Proceedings of theNational Academy of Sciences of the United States of America	2	12,779		2,61	13,45	805	83,36	1	113,5		16
Reproduction	1	3,923		0,87	4,322	142	78,21	1	158		2
Reproductive Biology and Endocrinology	2	4,982		1,08	5,426	95	66,78	2	157,5		10,5
Steroids	2	2,76		0,56	2,772	107	21,45	4	188		25
Trends in Endocrinology & Metabolism	1	10,586		1,4	14,206	178	93,49	1	124		3

When the studies were classified according to subtypes, 56 papers were original scientific papers with 193 (135.0-258.0) mean citations and a mean AS of 32.5 (15.3-52.7), whereas 44 papers were reviews and meta-analyses with 193 (150.6-258.0) mean citations and a mean AS of 16.0 (8.6-43.2) (Table 3).

**Table 3:** Altmetric scores and citation numbers of the Top 100 articles, ranked according to the study types Note: Median (25%-75% interquartile range) were used.

Study Type	Number of Articles	Altmetrics Scores	Citations
All article	100	17.6 (15.0-52.7)	193 (145.2-258.0)
Orginal scientific paper	56	32.5 (15.3-52.7)	193 (135.0-258.0)
Review	44	16.0 (8.6-43.2)	193 (150.6-258.0)

In 2012, 27 research papers were published with a mean AS of 17.6 (8.0-41.1) and a mean citation number of 193.7 (150.6-283.5). Although nine articles in the Top 100 list were published in 2018, the highest AS was 44.0 (11.5-168.0), and the average citation number was 158.5 (119.0-225.8) (Table 4).

**Table 4:** Altmetric scores and citation numbers of the Top 100 articles, ranked according to published years Note: Median (25%-75% interquartile range) were used. NA: not applicable.

Published years of articles	Number of articles	Altmetrics Scores	Citations
2012	27	17.6 (8.0-41.1)	193.7 (150.6-283.5)
2013	17	35.0 (16.3-52.7)	193.7 (155.0-193.7)
2014	11	16.0 (9.0-52.7)	193.7 (133.5-283.5)
2015	14	17.3 (15.0-48.8)	190.0 (132.6-208.0)
2016	13	17.6 (12.7-59.7)	258.0 (135.7-279.9)
2017	8	20.8 (15.8-51.5)	143.0 (126.0-193.7)
2018	9	44.0 (11.5-168.0)	158.5 (119.0-225.8)
2019	1	275.5 (NA)	275.5 (NA)

Correlation analysis results between the AS and IF, 5-year IF, H-index, total WoS citation number, Q category, JIF percentile and JCI value are illustrated in Table 5.

**Table 5:** Correlation between metrics Notes: IF: Impact Factor, JCI: Journal Citation Indicator, JIF: Journal Impact Factor, AS: Altmetrics Score, Q category: Quartile category The values above the diagonal consisting of 1 values extending from the top left to the bottom right represent the "r" value. The values below represent the "p" value. *Correlation is significant at the 0.01 level (2-tailed). **Correlation is significant at the 0.05 level (2-tailed).

	5-Year IF	IF	H Index	AS of Article	Citation Number of Article	Q category	JIF Percentile	Journal Citation Indicator (JCI)
5-Year IF	1	.989**	.428**	.168	.403**	-.685**	.930**	.846**
IF	.000	1	.437**	.187	.371**	-.689**	.925**	.837**
H Index	.000	.000	1	.536**	.313**	-.563**	.375**	.393**
AS of Article	.097	.064	.000	1	.416**	-.358**	.168	.172
Citation Number of Article	.000	.000	.002	.000	1	-.437**	.451**	.388**
Q category	.000	.000	.000	.000	.000	1	-.728**	-.641**
JIF Percentile	.000	.000	.000	.096	.000	.000	1	.852**
Journal Citation Indicator (JCI)	.000	.000	.000	.088	.000	.000	.000	1

The AS was positively correlated with H-index (r: 0.536, p <0.05), total WoS citation number of article (r: 0.416, p <0.05) and Q category (r: 0.358, p <0.05). There were no correlations with IF (r: 0.187, p =0.064), 5-year IF (r: 0.168, p =0.097), JIF percentile (r: 0.168, p =0.096), and JCI value (r: 0.172, p =0.088).

## Discussion

As far as we know, this is the first study to analyze the 100 most cited articles on the topic, title, and keywords of polycystic ovary syndrome or PCOS published in all journals in terms of traditional metrics and altmetrics.

The number of citations an article receives after it is published shows the contribution of the article to science and the effectiveness of the article. However, even if the scientific article is making significant contributions to science, authors citing this article in their new studies, the peer-review process, acceptance, and publication of the article may cause years to pass before the previous article is cited [11,12]. In addition, citations of some important studies will be affected because journals allow a specific order of references and authors use this limited order of references. However, nowadays, thousands of scientific contents are shared on the internet and respected journals ensure that accepted articles are first recognized online so that the scientific content is cited more. For this situation, many journals offer open access publication options and want the scientific article to be online very quickly. Using a variety of sources, altmetrics report the engagement level with research posted online using the daily activity of researchers on the internet and social media [13]. Because both the public and academics use altmetrics, they can be subjected to negative interferences (such as spam). An evaluation of the Top 100 trending articles in neurosurgery journals found that the articles in journals with social media accounts have a higher AS than articles in journals without a social media account [14]. Consequently, some journals aggressively use social media to promote their articles while others do not, which can greatly impact the AS. In addition, popular topics may receive a lot of interaction, whereas more technical topics may receive less interaction.

Another important factor affecting the coverage of the articles on social media may be whether they address important diseases affecting society. For example, today, the widespread news value of headlines such as COVID-19, obesity, and hypertension causes them to be more effective on social media. Of course, this does not apply to PCOS. Few studies evaluate PCOS-related research with bibliometric analysis [15,16]. In addition to these studies, as far as we know, this is the first study to analyze the 100 most cited articles on the topic, title, and keywords of polycystic ovary syndrome or PCOS published in all journals in terms of bibliometrics and altmetrics. When reviewing the articles on the Top 100, the studies with the highest AS are research articles and reviews on the diagnosis of PCOS and its relationship with infertility. When we evaluated the AS and citation numbers of the Top 100 articles ranked according to published years, 27 research papers were published in 2012 with a mean AS of 17.6 (8.0-41.1) and a mean citation number of 193.7 (150.6-283.5). Although 9 articles in the Top 100 list were published in 2018, the highest AS was 44.0 (11.5-168.0), and the average citation number was 158.5 (119.0-225.8). The increase in AS can be attributed to the increased usage of social media over the years. Because recently published articles have not had time to be frequently cited, they were excluded from our list and evaluation. In addition, we found that although there are similar citations, original scientific papers have a higher AS than systemic reviews and meta-analyses, which was not expected. In the current study, we found that the AS was positively correlated with H-index (r: 0.536, p <0.05), total WoS citation number of article (r: 0.416, p <0.05) and Q category (r: 0.358, p <0.05). There were no correlations with IF (r: 0.187, p =0.064), 5 years IF (r: 0.168, p =0.097), JIF percentile (r: 0.168, p =0.096), and JCI value (r: 0.172, p =0.088). Similarly, a study of urology journals found a weak correlation between the AS and the number of citations [17]. In radiology, the AS and the number of citations also showed a weak correlation [18].

Our study had some strengths. The AS for the 100 most-cited articles in the PCOS field was examined for the first time. In our study, we examined all medical journals instead of only gynecologic journals, which enhance the effectiveness of the study results.In addition, we scanned not only the topics of the study but also scanned the title and keywords. In this way, we did not miss many important studies. Additionally, open access journals were included indiscriminately when scanning. This will of course have an impact on AS. The AS can reflect attention at the moment for an article but not necessarily its true worth over time. However, good communication on social media can boost scientific productivity and impact society. For journals, altmetrics can be an important tool for reaching their targeted audience and letting them know how much attention an article has received. The AS is useful as a supporting metric and its importance is expected to increase over time.

## Conclusions

Our results suggest that the AS is related to article total WoS citation number, journal Q category and journal H-index but unlike traditional bibliometrics, it may be insufficient to determine an article’s overall scientific importance. However, effective communication on social media can promote scientific productivity and have a positive impact on society.
